# Lyophilized alginate-based microspheres containing *Lactobacillus fermentum* D12, an exopolysaccharides producer, contribute to the strain’s functionality in vitro

**DOI:** 10.1186/s12934-021-01575-6

**Published:** 2021-04-17

**Authors:** Katarina Butorac, Jasna Novak, Barbara Bellich, Lucrecia C. Terán, Martina Banić, Andreja Leboš Pavunc, Slaven Zjalić, Paola Cescutti, Jagoda Šušković, Blaženka Kos

**Affiliations:** 1 Laboratory of Antibiotic, Enzyme, Probiotic and Starter Culture Technologies, Faculty of Food Technology and Biotechnology, University of Zagreb, Pierottijeva 6, Zagreb, Croatia; 2 Department of Life Sciences, University of Trieste, Via Licio Giorgieri 1, Bdg. C11, 34127 Trieste, Italy; 3 Department of Ecology, Agronomy and Aquaculture, University of Zadar, Trg Kneza Višeslava 9, 23000 Zadar, Croatia

**Keywords:** *Eps* cluster, Exopolysaccharides, *Limosilactobacillus* (*Lactobacillus) fermentum*, Probiotic, Prebiotic

## Abstract

*Lactobacillus (Limosilactobacillus) fermentum* D12 is an exopolysaccharide (EPS) producing strain whose genome contains a putative *eps* operon*.* Whole-genome analysis of D12 was performed to disclose the essential genes correlated with activation of precursor molecules, elongation and export of the polysaccharide chain, and regulation of EPS synthesis. These included the genes required for EPS biosynthesis such as *epsA*, *B*, *C*, *D* and *E*, also *gt*, *wzx,* and *wzy* and those involved in the activation of the precursor molecules *galE*, *galT* and *galU*. Both the biosynthesis and export mechanism of EPS were proposed based on functional annotation. When grown on MRS broth with an additional 2% w/v glucose, *L. fermentum* D12 secreted up to 200 mg/L of a mixture of EPSs, whose porous structure was visualized by scanning electron microscopy (SEM). Structural information obtained by ^1^HNMR spectroscopy together with composition and linkage analyses, suggested the presence of at least two different EPSs, a branched heteropolysaccharide containing t-Glc*p* and 2,6-linked Gal*f*, and glycogen. Since recent reports showed that polysaccharides facilitate the probiotic-host interactions, we at first sought to evaluate the functional potential of *L. fermentum* D12. Strain D12 survived simulated gastrointestinal tract (GIT) conditions, exhibited antibacterial activity against enteropathogenic bacteria, adhered to Caco-2 cells in vitro, and as such showed potential for in vivo functionality. The EPS crude extract positively influenced D12 strain capacity to survive during freeze-drying and to adhere to extracellular matrix (ECM) proteins but did not interfere Caco-2 and mucin adherence when added at concentrations of 0.2, 0.5, and 1.0 mg/mL. Since the viable bacterial count of free D12 cells was 3 logarithmic units lower after the exposure to simulated GIT conditions than the initial count, the bacterial cells had been loaded into alginate for viability improvement. Microspheres of D12 cells, which were previously analyzed at SEM, significantly influenced their survival during freeze-drying and in simulated GIT conditions. Furthermore, the addition of the prebiotic substrates mannitol and lactulose improved the viability of *L. fermentum* D12 in freeze-dried alginate microspheres during 1-year storage at 4 °C compared to the control.

**Supplementary Information:**

The online version contains supplementary material available at 10.1186/s12934-021-01575-6.

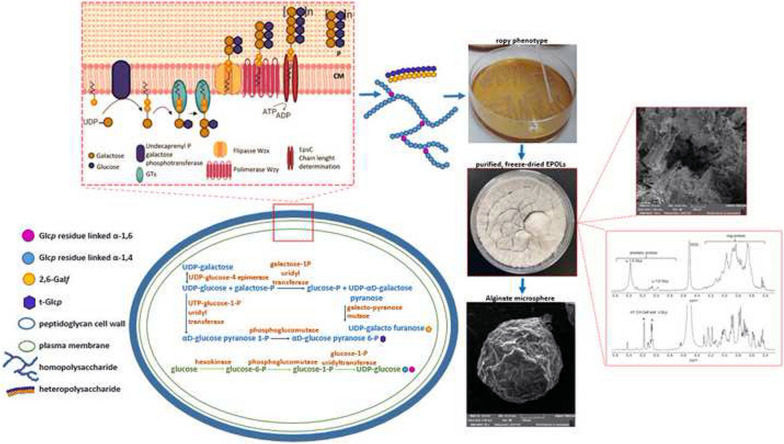

## Introduction

Exopolysaccharides (EPSs) are high-molecular-mass carbohydrate polymers produced by a diversity of microorganisms. Recently EPS derived from lactic acid bacteria (LAB) are gaining attention. Their physiological roles in the LAB cells are not yet fully understood. However, several authors hypothesized that EPSs ensure bacterial cell protection and mediate cell adhesion [1–3]. Accumulating research emphasizes the new, other than traditionally described functions of EPS produced by *Lactobacillus* strains, which contribute to cell-host interactions [4–7]. These molecular interactions can result in EPS lowering cholesterol and triglyceride levels [8], a hindering of the adhesion of pathogens [9], and exerting antitumor [10], antimicrobial, antiviral [6] and immunomodulatory activities [5, 11, 12]. Owning to the feasibility to be used as fermentable substrates for the desirable bacteria their potential prebiotic properties were also recognized [8, 13].

Microbial polysaccharides can be hetero- or homo-polysaccharides which are either attached to the bacterial cell surface or loosely secreted into the cell microenvironment [11]. Due to diversity in structures, the analysis of the associated biosynthesis clusters is quite challenging. Here we focused on the EPS produced by *Lactobacillus (Limosilactobacillus) fermentum* D12 which displays a characteristic ‘ropy’ phenotype after growth in MRS medium. To get new insights into the structure and properties of this EPS, we performed diverse structural analyses and supported the results by genome sequencing and subsequent analyses of strain probiotic properties which could be related to EPS biosynthesis. Furthermore, with the aim of optimizing the probiotic delivery system, we evaluated the efficiency of alginate microencapsulation, prebiotic supplementation and freeze-drying of *L. fermentum* D12 to improve the viability of probiotic cells during 1-year storage and gastrointestinal tract (GIT) passage with the controlled release of probiotic cells at the targeted site of action.

## Results

### Identification of gene cluster for exopolysaccharide (EPS) biosynthesis

**Fig. 1:**
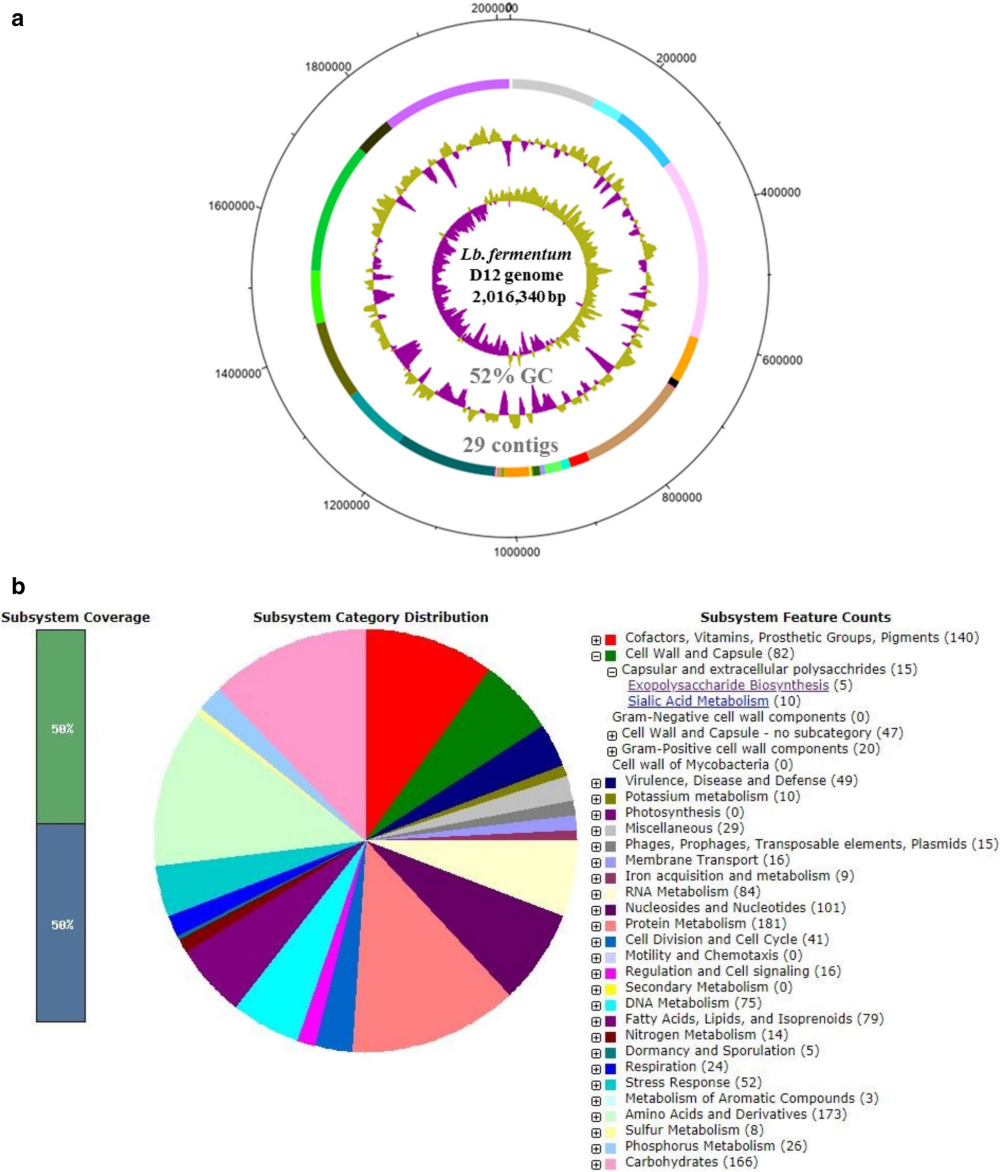
**a** Sequencing data were analyzed with DNA Plotter to construct the genomic atlas. Circles illustrate the following from outermost to innermost rings: (1) the entire chromosome; (2) the location of the contigs; (3) the local % GC plot and innermost, the GC-skew. **b** Subsystem distribution of *L. fermentum* D12 based on the RAST. The underlined text indicates the subsystem features involved in exopolysaccharide (EPS) biosynthesis

**Fig. 2:**
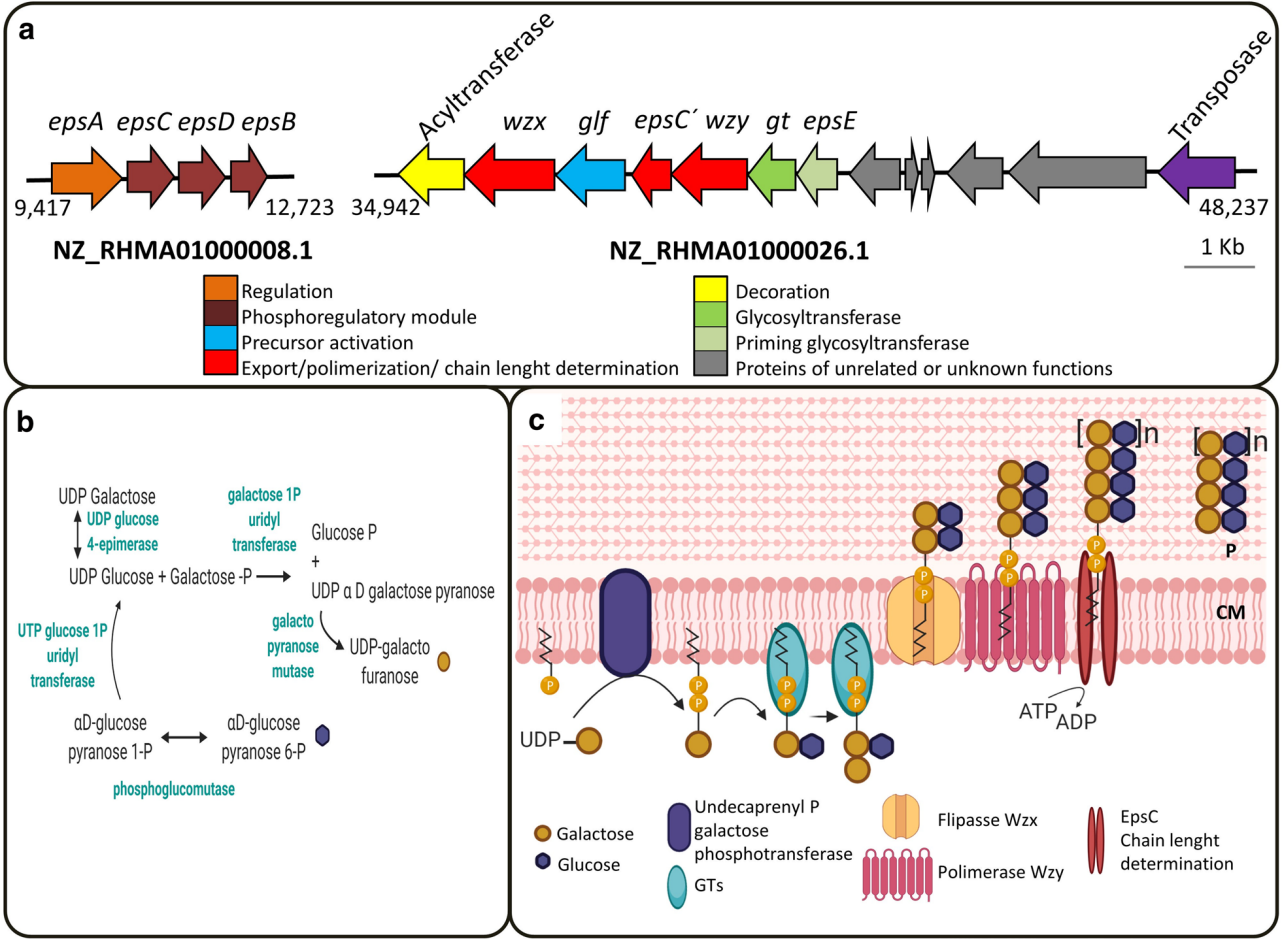
**a** Putative exopolysaccharide gene cluster (*eps*) located in contig NZ_RHMA01000026.1 and other genes involved in exopolysaccharide production located in contig NZ_RHMA01000008.1 by *L. fermentum* D12 strain. *epsA*: transcriptional regulator; *epsC*: tyrosine kinase modulator; *epsD*: tyrosine kinase; *epsB*: manganese-dependent tyrosine phosphatase; *wzx*: flippase; *glf*: UDP-galactopyranose mutase; *epsC*´: tyrosine kinase modulator/chain length determination; *wzy*: polysaccharide polymerase; *gt*: glycosyltransferase; *epsE*: Undecaprenyl galactose phosphotransferase; C: cytoplasmic membrane; P: periplasm. Transcribing directions, gene functions and module size are respectively shown by oriented arrows, different colors and arrow length. **b** Putative genes encoding the enzymes involved in the activation of the precursor molecules: glucose pyranose and galactose furanose. **c** Proposal of the putative biosynthetic model of the EPS cluster of *L. fermentum* D12. This image was created with Biorender.com

The draft genome sequence of *L. fermentum* D12, recently reclassified as *Limosilactobacillus fermentum* [14], consists of 2,016,340 nucleotide base pairs in 29 contigs (Fig. 1a). The G+C content of 52.0% together with the genome size of 2.02 Mb is consistent with the type strain ATCC 14931 [14] and with the well-described *L. fermentum* IFO 3956 [15]. The number of identified coding DNA sequences (CDS) is 1971 and the number of RNAs is 64. The phylogenetic position of *L. fermentum* D12 was determined from its whole genome sequence, relative to other publicly available *L. fermentum* sequenced genomes. The analysis highlighted a pairwise high similarity of D12 and KMB_613 strains, the latter isolated from Slovakian cheese [16], and both clustered close to the HFB3 strain (Additional file 1: Fig. S1). Detection of the putative EPS-encoding genes was one of the main goals of the whole genome sequencing (WGS) of *L. fermentum* D12. The genome sequence was annotated to in silico analyze the subsystem categories and detect the functions related to EPS biosynthesis (Fig. 1b). Genes potentially associated with EPS biosynthesis were identified (Fig. 2a).

The genome harbors seven genes coding for the phosphotransferase system (PTS) for sugar uptake. *L. fermentum* D12 genome includes genes related to the mannose-specific components of the phosphoenolpyruvate: PTS, but also sucrose and cellobiose. This correlates with the D12 strain ability to metabolize d-mannose, cellobiose, sucrose, d-glucose, d-galactose, d-lactose, d-ribose, d-xylose, d-fructose, maltose and melibiose as revealed by an analysis of sugar fermentation profiles with API 50 CHL (data not shown). Putative genes encoding the enzymes involved in the activation of the precursor molecules, necessary for the stepwise elongation of the EPS, were annotated (Additional file 6: Table S1). They include the genes *glf*, *galE*, *galT* and *galU* that encode for UDP-galactopyranose mutase, UDP-4-epimerase, galactose 1-*P*-uridyltransferase and UTP-1-glucose 1-phosphatase uridyltransferase, respectively (Additional file 6: Table S1, Fig. 2b). Also, the genes involved in the next steps of EPS synthesis are described below (Additional file 6: Table S1). The phosphoregulatory module is at the extreme of contig NZ_RHMA01000008.1 and includes the genes *epsC* (GW747_RS03540), *epsD* (GW747_RS03545) and *epsB* (GW747_RS03550) whose products participate in facilitating the polymerization and chain-length determination of the EPS (Fig. 2), as well as *epsA* (GW747_RS03535), the LytR family transcriptional regulator, which regulates the EPS synthesis. The genes coding for a flippase Wzx (GW747_RS07660) and a polysaccharide polymerase Wzy (GW747_RS07675), the proteins responsible for transport across the cell membrane and polymerization were detected in contig NZ_RHMA01000026.1. The presence of both genes suggests that *L. fermentum* D12 strain could secrete EPS through the Wzx/Wzy-dependent pathway. Sequence analysis showed the presence of 12 and 8 transmembrane domains for *wzx* and *wzy*, respectively, and a GC content of 45.0% for *wzx* and 46.0% for *wzy*, both values lower than the one of the genome (52.0%), characteristics in agreement with those described for these genes among other lactobacilli [17]. The gene *epsC*´ (GW747_RS07670) whose functions are related to tyrosine kinase and chain length determination was also found. The priming glycosyltransferase, *epsE* (GW747_RS07685), corresponding to the undecaprenyl galactose phosphotransferase, as well as GW747_RS07680 gene, that encodes a protein containing a domain DUF4422 and is related with the incorporation of galactofuranose into polysaccharides, were also detected within the cluster. Detection of the genes involved in EPS production allowed us to propose a putative biosynthetic model of the *eps* cluster of *L. fermentum* D12 (Fig. 2c). A comparison between the genetic organization of this *eps* gene cluster with other clusters sharing the same type of organization [17] in other strains of *L. fermentum* CECT5716 (CP002033.1), IFO3956 (AP008937.1), SNUV175 (CP019030.1) and F6 (CP005958.1) is shown in Additional file 2: Fig. S2.

### Isolation and purification of exopolysaccharides (EPS) biosynthesised by *L. fermentum* D12

**Fig. 3:**
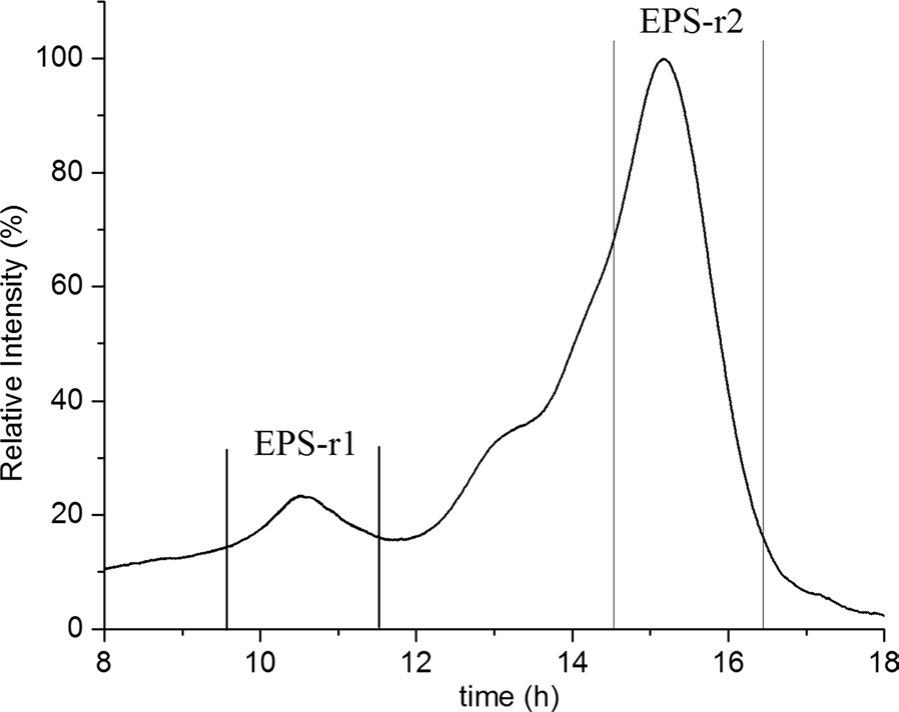
Size-exclusion chromatography elution profile of EPS-r separated on a Sephacryl S-400 column. Fractions were pooled as shown

Several *L. fermentum* strains produce EPS, but the repeating unit structures of EPS from only three *L. fermentum* were described so far [7]. Direct macroscopic observation of the smooth and glistening D12 colonies growing on the surface of MRS agar medium showed a “ropy” phenotype characterised by the formation of a long filament when the colony is touched (Additional file 3: Fig. S3a). In order to differentiate the EPS released (EPS-r) into the culture medium from the EPS bound (EPS-b) to the cell surface, the yields of the EPSs (Y_EPS_) were determined in both fractions. According to Bukola et al*.* [18], active producers of EPSs are those strains whose yield of EPS production is above 40 mg/L. The yield of the EPS-r fraction was above 40 mg/L, which makes *L. fermentum* D12 an active producer of EPS, while the EPS-b fractions were recovered in very low yield (0.6–2.2 mg/L) and therefore they were not used for further analysis. Considering that the EPSs production can be induced by an excess of carbohydrates, *L. fermentum* D12 was grown in the MRS medium supplemented with one of the following sugars: glucose, galactose, lactose, fructose or sucrose. When using glucose as a carbon and energy source, D12 strain produced the highest amount of 200 mg/L EPS-r. The yields of the EPSs released into the culture media (EPS-r) enriched with fructose, lactose, sucrose and galactose, were 193 mg/L, 181 mg/L, 167 mg/L and 165 mg/L, respectively. ^1^H NMR spectra were recorded for all EPSs-r obtained with different carbon sources and they indicated no difference among the different EPSs-r samples (data not shown). Our results are consistent with those of van den Berg et al. [19] who showed that the addition of the different carbon sources such as fructose, lactose, galactose did not affect the structure of the *Lactobacillus sake* 0–1 EPS. Therefore, the EPS extracts were pooled together, purified from proteins by TCA precipitation, followed by ethanol precipitation, centrifugation, dialysis and filtration. The EPS-r solution was taken to pH 6.7 before lyophilisation and 236 mg of polysaccharide was obtained. D12-EPS secreted to the environment, was visualized using SEM (Additional file 3: Fig. S3b). EPS-r was subjected to low-pressure size exclusion chromatography on a Sephacryl S-400 column. The elution profile showed two peaks and a shoulder; the fractions corresponding to the two peaks were pooled together (Fig. 3), and named EPS-r1 and EPS-r2, while the shoulder fractions were left apart, because their ^1^H NMR spectrum showed the presence of manno-polymers most likely derived from yeast extract contained in MRS broth (data not shown). The ratio between EPS-r1 and EPS-r2 was calculated from their peak area and resulted to be 1:12.

### Structural characterization of D12 EPS-r

**Fig. 4.**
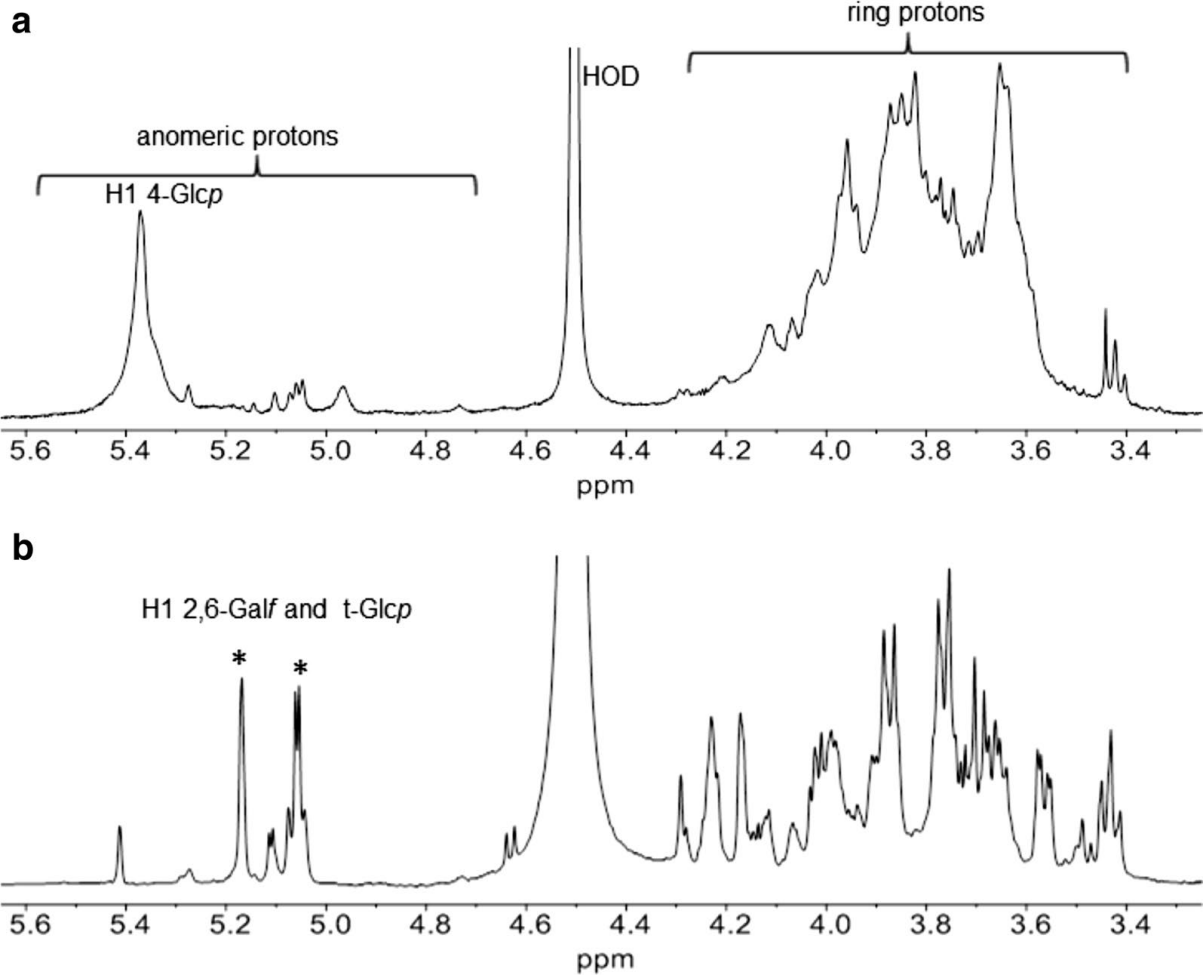
^1^H NMR spectra of EPS-r1 (**a**) and EPS-r2 (**b**) in D_2_O recorded at 500 MHz and 50 °C. **a** α-1,4 Glc*p* and α-1,6 Glc*p* = anomeric protons of 1,4- and 1,6-linked glucopyranose; **b** in the EPS-r2 spectrum the stars indicate the two most intense anomeric proton resonances attributed to t-Glc*p* and 2,6-Gal*f*

The EPS-r1 and EPS-r2 samples were subjected to ^1^H NMR spectroscopy which showed spectra typical of polysaccharides; they were also different from each other, indicating production of at least two different polymers by *L. fermentum* D12 (Fig. 3). The ^1^H NMR spectrum of EPS-r1 showed one main resonance in the anomeric region at 5.37 ppm (Fig. 4a) indicative of sugar in the α-anomeric configuration, thus suggesting that the main component of this fraction is a homopolymer. Linkage analysis revealed 4-linked Glc*p* as the main monosaccharide of EPS-r1, together with small amounts of 4,6-linked Glc*p*, suggesting the presence of a branched glucan; integration of their peak area indicated 10% of branching. Comparison of EPS-r1 ^1^H NMR spectrum with that of published data [20, 21] led to identify it with glycogen. Apart from the resonance at 4.96 ppm which belongs to 1–6 linked Glc. The other low-intensity signals in the ^1^H NMR spectrum of EPS-r1 most likely belong to other molecules, still present despite the extensive purification procedure applied.

The anomeric region of the EPS-r2 ^1^H NMR spectrum (Fig. 4b) showed two main resonances at 5.18 and 5.07 ppm with identical integration value of their peak areas, and five less intense peaks at 5.42, 5.12, 5.08, 5.05 and 4.64 ppm, suggesting the presence of one main polysaccharide constituted of two different sugars in the α-anomeric configuration together with one or more other polymers. Unfortunately, because of the tendency to form inter-chain hydrogen bonds, sometimes polysaccharides are difficult to obtain in a pure state. Composition analysis revealed Man:Gal:Glc in 0.4:1.0:0.9 relative molar ratios, suggesting that the two major anomeric peaks in the ^1^H NMR spectrum belong to Gal and Glc, while Man is probably due to small amounts of manno-proteins from yeast extract still present after the purification procedure applied. Linkage analysis showed two main residues terminal non-reducing Glc*p* (t-Glc*p*) and 2,6-linked Gal*f* in the molar ratios of 1.0:0.5, thus suggesting that EPS-r2 is a heteropolysaccharide composed of a branched disaccharide repeating unit with 2,6-α-Gal*f* in the backbone and α-Glc*p* in the side chain. The amount of 2,6-Gal*f* detected by linkage analysis is lower than expected from NMR data and this might be due to the less stable furanose configuration of the Gal residue with respect to the pyranose one of the glucose.

In conclusion, *L. fermentum* D12 strain produces and releases two different polysaccharides in the growth conditions applied: a branched heteropolysaccharide containing t-Glc*p* and 2,6-linked Gal*f*, and glycogen, the latter being present in a smaller quantity, 1/12 of the former. The branched EPS is different from the three structures already reported in the literature for other *L. fermentum* strains [7].

### In vitro functionality of *L. fermentum* D12 strain

In the context of antibiotic resistance, it is essential to define the antibiotic susceptibility of the candidate strains before further evaluation as probiotics. *L. fermentum* D12 was susceptible to ampicillin, chloramphenicol, clindamycin, erythromycin, gentamicin, tetracycline, but not to vancomycin, kanamycin, streptomycin (data not shown) based on the minimum inhibitory concentration breakpoints (MIC) established by the European Food Safety Association [22]. Other investigators have reported lack of susceptibility to different antibiotics in *Lactobacillus* strains, where an intrinsic vancomycin-resistant phenotype is the best-characterized while an intrinsic resistance to aminoglycosides is speculated to be related to the lack of cytochrome-mediated drug transport [23].

**Fig. 5:**
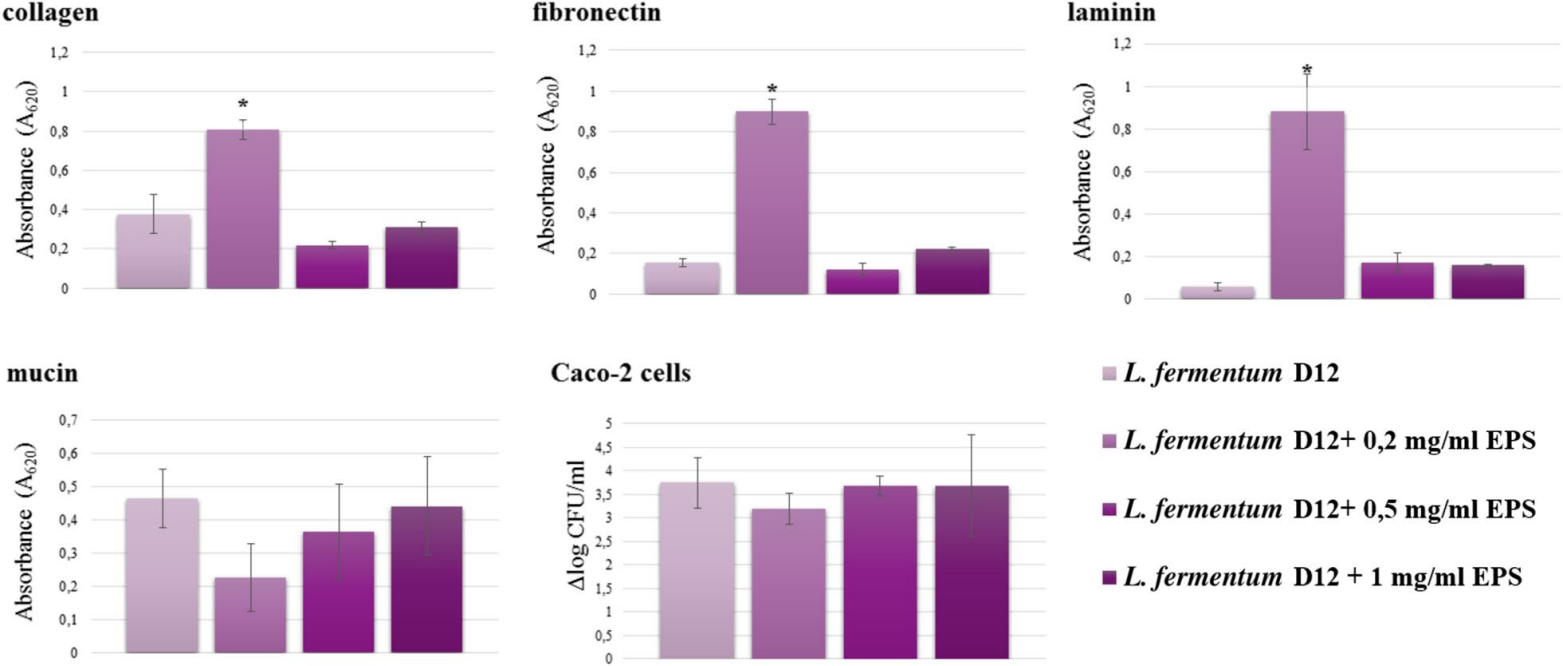
Adhesion ability of *L. fermentum* D12 to major EMC proteins, mucin and Caco-2 cells in the presence of increasing concentrations of the EPS fractions. *Significantly different (P < 0.01) from other samples

Another aim was to examine whether EPSs have a natural protective effect on the producer strain under stressful conditions such as lyophilization and to compare whether the same effect of EPS is exerted on the non-EPS producing strain *L. plantarum* D13. The lyoprotective effect of D12 EPS was assayed by adding different EPS concentrations (0.2, 0.5 and 1.0 mg/mL) before exposure of D12 and D13 strains to freeze-drying (Table 1a). *L. plantarum* D13 has been previously used as a control strain, as it is from the same microenvironment as the D12 strain [24, 25]. Its probiotic properties are defined and the whole genome is available (GenBank Accession number: NIGG00000000). The highest tested concentration of EPS was 5 times higher than the concentration produced by the D12 strain during growth (0.2 mg/mL), which was added as the lowest tested concentration. Selected common lyoprotectants such as lactose, sucrose, sorbitol or inulin were added at a concentration 100 times higher than the maximum added concentration of D12 EPS, to assess their lyoprotectant capacities. Their protective effect was significantly higher than that of EPS (Table 1b). However, the results indicated a positive impact of EPS on D12 cell survival with significantly better survival at increasing EPS concentrations, suggesting EPS capacity to protect cells during freeze-drying (Table 1a). Although the results indicated significantly higher survival capacities of the D13 compared to the D12 strain, when tested in the range of added concentrations of D12 EPS (0–1 mg/mL), the difference in mortality between the two strains during freeze-drying was less than 1 Δlog CFU/mL (Table 1a). Moreover, the results indicated highly similar survival rates for D13, when D12 EPS was added or not, suggesting EPS did not play a role in its survival during freeze-drying. In addition to protecting the cells, the EPSs’ influence on D12 cell adhesion to intestinal mucosal and epithelial surfaces has also been examined. Therefore, considering that the viability of D12 cells after the passaging through the simulated GIT conditions was above 10^6^ CFU/mL with the capacity of 65.75 ± 3.44% to adhere to colonocyte-like Caco-2 cell line (Fig. 5), we further assessed *L. fermentum* D12 binding capacity to major extracellular matrix (ECM) proteins laminin, collagen, fibronectin; glycoprotein mucin and Caco-2 cells in the presence of crude EPS extract in different concentrations (0.2, 0.5 and 1.0 mg/mL). The adhesion levels varied depending on EPS concentration (Fig. 5). Namely, EPS crude extract significantly promoted adhesion to ECM proteins at the concentration of 0.2 mg/mL, whereas higher EPS concentrations (0.5 mg/mL and 1 mg/mL) did not significantly affect the adherence capacity of D12 cells compared with the control. In contrast, exogenous D12 EPS reduced the adhesion capacity of *L. plantarum* D13 (Additional file 4: Fig. S4). There is a possibility that EPS could directly adhere to the intestinal surfaces and interfere with bacterial adhesion, or they could stick to the cell surface and thereby shield adhesion factors.

**Table 1:** Survival of *L. fermentum* D12 and *L. plantarum* D13 after freeze-drying. (a) Influence of exopolysaccharide (EPS) addition and microencapsulation in alginate (b) Influence of the addition of lyoprotectants

*Lactobacillus* strain	Cell survival	EPS concentration (mg/mL)				Encapsulation in alginate
		0	0.2	0.5	1.0	
(a)						
D12	Before freeze-drying (log CFU/g)	9.77 ± 0.21^az^	9.99 ± 0.29^az^	9.79 ± 0.14^az^	9.62 ± 0.01^az^	10.13 ± 0.12^az^
	After freeze-drying (log CFU/g)	8.19 ± 0.19^bx^	8.79 ± 0.17^by^	8.68 ± 0.09^by^	8.69 ± 0.09^by^	9.98 ± 0.04^az^
	Cell mortality after freeze-drying (Δlog CFU/g)	1.57 ± 0.19^cx^	1.20 ± 0.17^cxy^	1.11 ± 0.09^cy^	0.92 ± 0.09^cy^	0.15 ± 0.04^bz^
D13*	Before freeze-drying (log CFU/g)	9.91 ± 0.01^az^	9.99 ± 0.04^az^	9.83 ± 0.06^az^	9.91 ± 0.07^dz^	10.05 ± 0.23^az^
	After freeze-drying (log CFU/g)	9.33 ± 0.05^dy^	9.16 ± 0.15^by^	8.99 ± 0.03^dy^	9.12 ± 0.19^by^	8.39 ± 0.35^cz^
	Cell mortality after freeze-drying (Δlog CFU/g)	0.58 ± 0.05^ex^	0.83 ± 0.15^cy^	0.84 ± 0.03^ey^	0.79 ± 0.19^cxy^	1.65 ± 0.35^dz^

Strain	Cell survival	Lyoprotectants (10% (w/v))			
		Lactose	Inulin	Sorbitol	Sucrose
(b)					
D12	Before freeze-drying (log CFU/g)	10.21 ± 0.20^az^	10.21 ± 0.20^az^	10.21 ± 0.20^az^	10.21 ± 0.20^az^
	After freeze-drying (log CFU/g)	10.09 ± 0.13^az^	9.90 ± 0.24^az^	10.08 ± 0.18^az^	9.85 ± 0.11^az^
	Cell mortality after freeze-drying (Δlog CFU/g)	0.23 ± 0.15^bz^	0.30 ± 0.15^bz^	0.12 ± 0.15^bz^	0.35 ± 0.11^bz^
D13*	Before freeze-drying (log CFU/g)	10.18 ± 0.15^az^	10.18 ± 0.15^az^	10.18 ± 0.15^az^	10.18 ± 0.15^az^
	After freeze-drying (log CFU/g)	10.08 ± 0.13^ay^	10.02 ± 0.18^ay^	9.64 ± 0.04^cz^	9.81 ± 0.49^ay^
	Cell mortality after freeze-drying (Δlog CFU/g)	0.09 ± 0.11^by^	0.16 ± 0.17^by^	0.54 ± 0.04^dz^	0.37 ± 0.49^yz^

^abcde^Different letter means statistically significant difference (*p* < 0.05) within the same column ^xyz^Different letter means statistically significant difference (*p* < 0.05) within the same row. The analysis was carried out using ANOVA and the results are reported as mean value ± SD of three independent experiments

^*^L. plantarum D13 represents EPS-non producing strain

Furthermore, there was no significant influence on the adhesion of *L. fermentum* D12 to mucin and Caco-2 intestinal epithelial cells in the presence of D12 EPS concentrations in comparison to the control. However, the genome sequence of the D12 strain predicts the expression of mucus- and fibronectin-binding proteins that could be involved in bacterial adhesion to the GIT. Thereby, probably proteins act as epithelial cell adhesins or ECM binding proteins.

Adhesion ability to intestinal cells can be associated with the potential to exclude pathogen species. *Lactobacillus* strains display a wide spectrum of antimicrobial activities established through the inhibitory action of the range of metabolites, including lactic acid, but also specific biomolecules like bacteriocins [26]. Therefore, in order to evaluate the potential to antagonize the infection of undesirable bacteria in the gut, the antimicrobial activity of *L. fermentum* D12 expressed as an effective inhibition ratio (EIR), against *Listeria monocytogenes* ATCC^®^19111™ and *Staphylococcus aureus* 3048 was tested. *L. fermentum* D12 exerted slightly lower inhibitory activity towards *L. monocytogenes* ATCC^®^19111™ (EIR = 1.312) than towards *S. aureus* 3048 (EIR = 1.875) using the soft agar overlay assay.

### Alginate microspheres for *L. fermentum* D12 gastrointestinal delivery

**Fig. 6:**
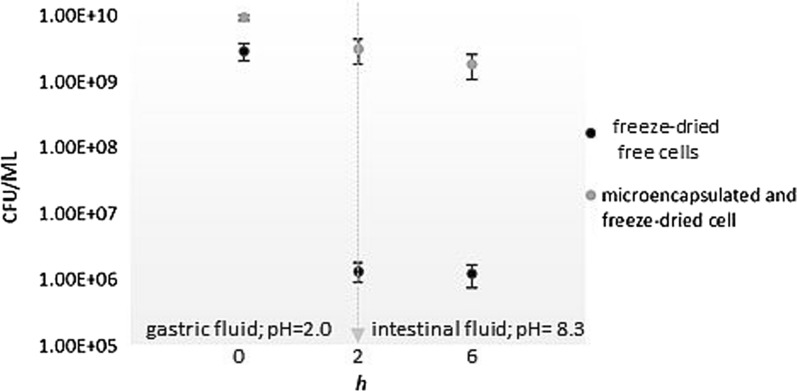
Viability of freeze-dried alginate-microspheres of cells (gray filled circle), and freeze-dried free cells (black filled circle) of *L. fermentum* D12, exposed to simulated GIT conditions (arrow indicates changed conditions: after 2 h incubation in simulated gastric fluid at pH = 2.0; 4 h incubation in the simulated intestinal fluid at pH = 8.3 was performed). Results are expressed as mean values of three independent experiments and error bars represent standard deviations

Orally consumed probiotic bacteria face several obstacles during GIT transit. These include extremely low pH, competition for nutrients and mucosal adhesion sites, effectors of the host immune system, all of which can influence the concentration of live microorganisms in situ. Therefore, we assayed the capacity of *L. fermentum* D12 to withstand GIT conditions by assessing its susceptibility to simulated gastric and intestinal fluids. The D12 strain withstood pH = 2 of gastric juice and the presence of the bile salts and pancreatin in the simulated small intestine conditions reaching the viable cell counts of 1.35 (± 0.43)·10^6^ and 1.22 (± 0.46)·10^6^ CFU/mL, respectively. The viable bacterial count of D12 cells was 3 logarithmic units lower after the exposure to simulated GIT conditions than the initial number of the cells (Fig. 6). Further, we performed microencapsulation in alginate with the aim to increase viable cell counts after freeze-drying, storage, and GIT transit, even if free cells of the D12 strain already demonstrated a survival rate above 10^6^ CFU/mL in simulated GIT conditions, a value considered sufficient for in vivo functionality with an initial daily dose of above 10^9^ CFU/mL [27]. SEM observations demonstrated that the produced alginate entrapped D12 cells were spherical (Additional file 5: Fig. S5), as a favorable shape for mass transfer of substrates. Namely, the morphology of the microcapsule affects capsule performance, which is important for mass transport behaviour [28]. The microencapsulation efficiency of 98.108 ± 0.485% of the initial D12 cell count in microspheres suggested that alginate is an appropriate matrix for encapsulation (Table 1a). Alginate microencapsulation positively influenced cell survival during lyophilisation (Δlog CFU/mL = 0.153 ± 0.042), while survival of free cells after freeze-drying was significantly lower (Δlog CFU/mL = 1.572 ± 0.275), implicating the protective effect of alginate microencapsulation (Table 1a). Considering the promising outcome of the alginate microencapsulation, thus prepared D12 cells were subjected to the simulated GIT conditions (Fig. 6). The survival of lyophilized cells entrapped in alginate and freeze-dried free cells significantly differed (P < 0.01). Cell mortality of free viable cells in simulated gastric fluid and small intestinal fluid (3.337 ± 0.155 and 3.384 ± 0.153 log CFU/mL, respectively) was significantly higher than cell mortality of microencapsulated D12 cells, 0.504 ± 0.162 and 0.737 ± 0.210 log CFU/mL, respectively (Fig. 6).

### Prebiotic impact on survival of *L. fermentum* D12 strain during storage

**Fig. 7:**
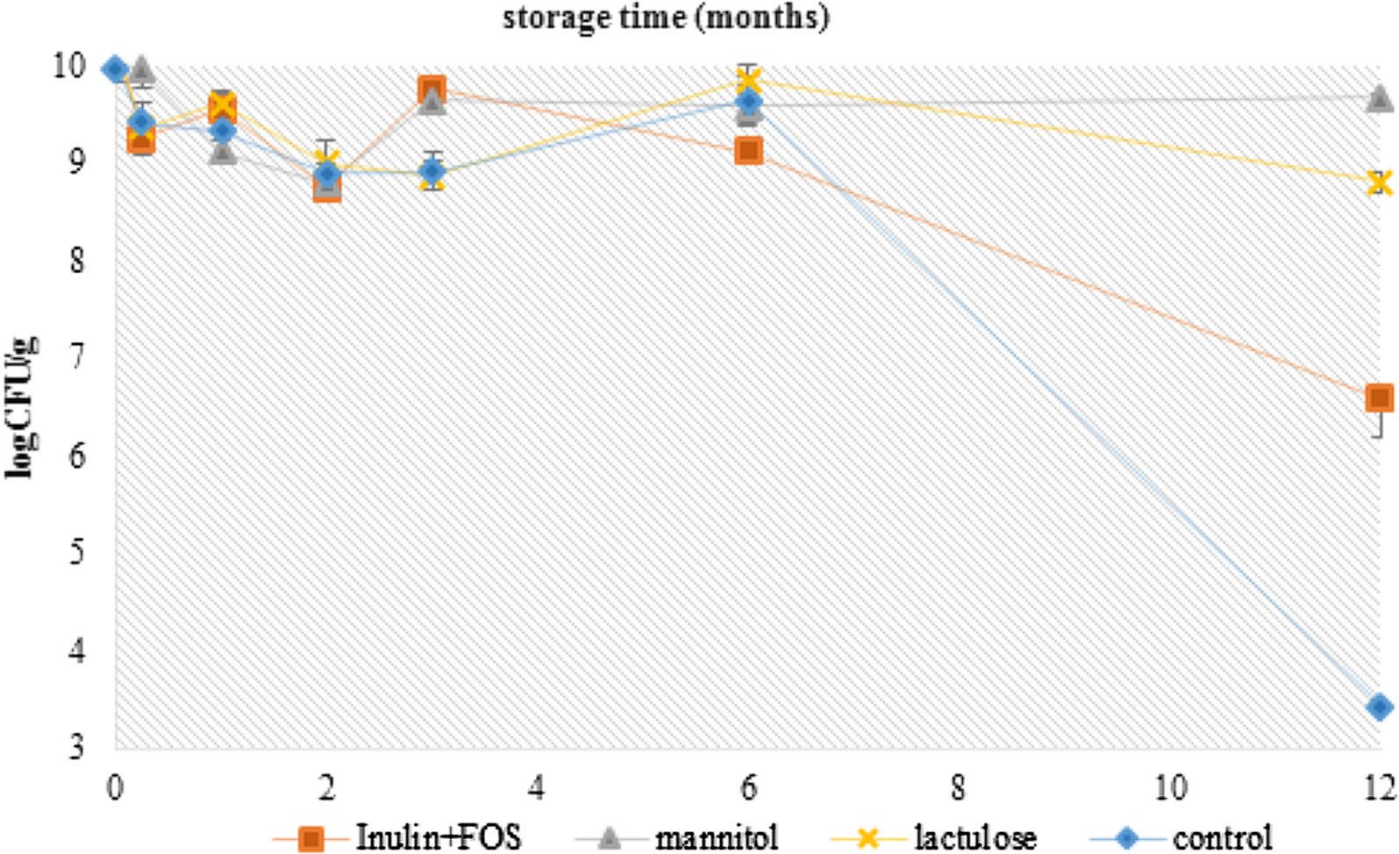
Survival of alginate microencapsulated *L. fermentum* D12 cells with prebiotics, during 1-year storage at 4 °C with the addition of different prebiotics: inulin with fructooligosaccharides (FOS), mannitol, lactulose; and without prebiotics. The results are expressed as mean values of three independent experiments and error bars represent standard deviations

Delivery of the maximal number of viable *L. fermentum* D12 cells to the colon is decisive to its functionality. Sustaining the viability of probiotic cells during processing and storage is crucial [29, 30]. Follow-up experiments included the microencapsulation of *L. fermentum* D12 bacterial cells in alginate with the addition of different prebiotic substrates, to assess the potential of protecting cells during storage and subsequently the delivery of sufficient viable cells to the colon. Three different prebiotic substrates, inulin in combination with fructooligosaccharides (FOS), mannitol and lactulose were applied during the microencapsulation process. The number of live D12 cells in the microspheres was maintained high over 6 months of storage at 4 °C, independently from the presence and type of prebiotic used (Fig. 7). However, a prebiotic-type-dependent significant reduction in the bacterial counts was observed after one year of storage at 4 °C. As shown in Fig. 7, approximately 3.437 ± 0.056 log CFU/mL of microencapsulated *L. fermentum* D12 without prebiotics survived after 1-year of storage at 4 °C, which resulted in a nearly 6 log loss of viable D12 cells. The viability of lyophilized D12 cell microencapsulated in alginate, supplemented with mannitol or lactulose, was merely affected since cell mortality was only 0.365 ± 0.032 log CFU/mL and 0.402 ± 0.047 log CFU/mL, respectively during the 1-year storage period. The cell mortality of *L. fermentum* D12 was significantly higher if inulin in combination with FOS was applied since the viable counts reduced from 10.259 ± 0.227 to 6.577 ± 0.396 log CFU/mL (Fig. 7). D12 strain metabolized mannitol, but not inulin according to API 50 CHL test (data not shown), which may indirectly contribute to cell resistance to stress conditions such as dehydration and long-term storage.

## Discussion

The newly identified *L. fermentum* D12 exhibited the ropy phenotype typical of EPS production. Strain D12 synthesized rather high amounts of crude EPSs, which were comparable to other reported EPS producers of the *Lactobacillus* genus [5, 31]. The natural production of EPS by LAB is relatively low (50–400 mg/L) compared to the production of EPSs by other species like 10–25 g/L of xanthan produced by the Gram-negative *Xanthomonas campestris* [32]. *L. fermentum* D12 could utilize 5 types of sugars as carbon and energy sources for both biomass formation and EPS synthesis. *L. fermentum* D12 secreted up to 200 mg/L of EPSs when grown in MRS broth with an additional 2% w/v of glucose which stimulated higher EPS production than the addition of galactose, lactose, fructose and sucrose in the same concentration. The influence of carbon sources on EPS production is also reported by other authors. In general, all LAB follow a conserved basic pathway for EPS synthesis which includes complex regulation mechanisms, chain length determination, biosynthesis and polymerisation of repeating units. Besides, EPS biosynthesis is coordinated by the mutual activity of genes involved in different cellular functions. Cultivation conditions, such as enrichment in specific sugar sources, can influence the amount of EPS synthesized, as well as the final product’s molecular structure and, consequently, their bioactive functions [8]. Noteworthy, these impacts on EPS composition are strain-dependent [33]. Despite it has been described that medium composition and growth conditions can affect the EPS composition [34], our results are consistent with those of van der Berg et al. [19] as the different sugars added to the medium did not affect the structure of the EPS, although they had an influence on the amount of EPS produced. Moreover, Roca et al. [33] described that, although the media and cultivation conditions can be manipulated to alter the composition of the EPS in several strains, for most bacteria, the EPS sugar composition is a genetically determined trait that is not notably affected by the cultivation conditions.

The optimal carbon source for EPS synthesis was glucose which may be partly attributed to the genes coding for enzymes of glucose transportation in *L. fermentum* D12 genome. Genome annotation revealed that D12 harbors a predicted *eps* cluster responsible for the production of EPS. This cluster includes the required set of genes described by Deo et al. [17] in different LAB for EPS biosynthesis, transport and regulation, comprising the presence of a priming glycosyltransferase *epsE*, another glycosyltransferase (*gt*), a flippase (*wzx*), a polymerase (*wzy*) and a tyrosine kinase (*epsC*´). However, the cluster lacks the gene *epsB* that encodes for the tyrosine kinase modulator, which might be compensated by the one in the phosphoregulatory module in contig NZ_RHMA01000008.1. In fact, as reported for *Lactobacillus plantarum* WCFS1 [35] which has more than one *eps* cluster, the lack of a required gene can be compensated by genes in other clusters. The absence of essential genes has been previously described for other lactobacilli genomes, where the most frequent missing genes were *wzy*, *epsB* and *epsC* (or both), *wzx* and *epsE* [17]. Other genes involved in the EPS production or the precursors’ activation can be found in other locations of the genome or be dispensable, like the acyltransferase (GW747_RS07655) located at the beginning of the cluster whose function includes the chemical decoration of the EPS, and also the genes *epsA* and *epsD* which are located in the contig NZ_RHMA01000008.1. It is interesting to point out the gene encoding for a transposase (GW747_RS07715) flanking the *eps* cluster, a characteristic also found in other lactobacilli [17]. Noteworthy, downstream of the *eps* cluster there are two glycosyltransferases (GW747_RS07505 and GW747_RS07510) which might also be involved in EPS production because they belong to family 4 and thus act with a retaining mechanism, producing α-anomers in agreement with the monosaccharide anomeric configuration found in the present study. Genes coding for galactofuranose biosynthesis and activation, and glucose activation were found, and they are in agreement with the composition of the two EPSs isolated from *L. fermentum* D12. They comprise the gene encoding the UDP-galactopyranose mutase (GW747_RS07665) which is the most common gene of sugars activation found in LAB [17]. The genetic organization of *eps* clusters in lactobacilli was classified by Deo et al. [17] into eight groups according to the presence and absence of genes through hierarchical clustering analysis. Some of these groups were termed “generic” which indicates the presence of *epsABCDE* genes at the 5′ end, as in the case of the well-described strain of *L. fermentum* YL-11 [36]; other *eps* clusters lacking the stretch *epsABCDE*, either because some of these genes are in a different position in the EPS cluster or are absent, were classified as “non-generic”. According to the sequence alignment, the organization of the *eps* cluster of *L. fermentum* D12 can be classified as “non-generic” and belonging to group 8 (Additional file 2: Fig. S2).

Expression of *eps* genes was proved by the isolation of polysaccharides from *L. fermentum* D12 culture. The EPSs porous structure was visualized by SEM after their isolation and purification from the growth medium of *L. fermentum* D12. Their primary structure was characterized by using composition and linkage analysis and ^1^H NMR spectroscopy. The results showed that D12 produces two EPSs: glycogen and a branched polysaccharide containing 2,6-Gal*f* in the backbone and t-Glc*p* in the side chain. The complete structure of the latter could not be defined because of the presence of other compounds, which rendered uncertain the acquisition of any further structural detail. In order to establish the complete primary structure of the branched polysaccharide, different growth media will be used since they may lead to selective or at least increased production of the polymer of interest.

The identification of *eps* cluster and isolation of two EPSs encouraged us to investigate if D12 strain has functionality potential. Besides protection of cells, some of the beneficial actions of EPS may be related to the formation of a layer surrounding the producer cells, which is involved in protection against pathogens or with immunostimulating and adhesion properties [3–6]. LAB-EPSs could stimulate the growth of bifidobacteria in the GIT in the same way as other prebiotics [13, 37]. Therefore, besides the suggested protective role of EPSs in the persistence of the D12 in the simulated GIT, there is also a potential to protect the cells during stress conditions like freeze-drying. EPSs surround the bacterial cells and can generate a highly hydrated boundary against desiccation effects. D12 also demonstrated a potential antagonistic effect against the enteropathogens *L. monocytogenes* ATCC^®^19111™ and *S. aureus* 3048, a characteristic important for both competitive exclusion and adhesion capacity in the GIT. Since the adhesion of probiotic bacteria could increase their persistence in the intestinal tract and enable close contact with the host, we further investigated *L. fermentum* D12 binding capacity to the intestinal epithelial cells, mucin, and ECM proteins and showed that it adhered strongly to enterocyte-like Caco-2 cells and had the binding capacity to collagen and mucin. According to the results, D12 EPS may influence the adherence capacity of *L. fermentum* D12, the effect being dose-dependent. At the concentration of 0.2 mg/mL of EPS, adhesion of D12 cells to ECM proteins was significantly promoted whereas 0.5 and 1 mg/mL of EPS did not influence the adherence capacity to ECM proteins compared with the control. According to the results, it is unlikely to increase the adhesion level of *L. plantarum* D13 with D12 EPS addition. Ruas-Madiedo et al. [38] demonstrated that the EPS fractions NB667 and IPLA-R1 significantly reduced the adherence of *B. animalis* IPLA-R1 and *L. rhamnosus* LGG. However, the addition of crude extract of EPS did not impair the adhesion of D12 cells to the Caco-2 cell line. These results suggest that the different structural characteristics of the EPS may account for their differential effect in bacterial cell adhesion. The EPS impact could be to inhibit the adhesion of competitive bacteria to intestinal sites, or it could stick to the bacterial cells and thereby mask the surface molecules involved in the adhesion of EPS producing strain. EPS production affects the adhesion ability of *Lactobacillus johnsonii*, *Lactobacillus rhamnosus* GG and *Lactobacillus reuteri* studied in different in vitro models [2, 38, 39]. The possible mechanisms of EPS influence of *L. fermentum* D12 cell adhesion on ECM proteins must be further examined.

Microencapsulation of probiotic cells in alginate is gaining attention due to the possibility to protect the microbial cell with the possibility of gradual release [40]. Alginate has convenient biochemical features and excellent mucoadhesive properties and therefore represents an attractive approach for in situ delivery of probiotic cells [25]. We used an alginate encapsulation strategy to shield *L. fermentum* D12 from adverse conditions, such as those during freeze-drying or during the transit of GIT. Morphology analysis of the microspheres by using SEM revealed that they were spherical. The survival of D12 cells microencapsulated in alginate was significantly higher than the survival of free cells during lyophilisation which confirms the protective effect of microencapsulation in alginate on *L. fermentum* D12. Furthermore, microencapsulated D12 cells survived in higher counts than free cells under simulated GIT conditions. Our observations are in accordance with the results of other authors, who reported about the protection role of microencapsulation in alginate during the gastrointestinal delivery of probiotic strains [41–43].

With the aim to further improve the viability of *L. fermentum* D12 cells in freeze-dried alginate microspheres, we also applied different prebiotic substrates. Besides the potential to modulate the gut microbiota in vivo, prebiotics have the potential to act as lyoprotectants which contribute to maintaining probiotic counts in functional food or biotherapeutic preparations. The minimum number of probiotic microorganisms imparting health benefits should be at least 10^9^ CFU per day, while for health-promoting effects in specific conditions a dose of at least 10^10^ CFUs per day is required [27]. Here we proved an outstanding survival rate of alginate-based lyophilized microspheres of D12 strain with the addition of mannitol and lactulose (ca 10^9^ CFU/g), while the survival in the presence of inulin with FOS was significantly lower after one-year storage (ca 10^6^ CFU/g). These findings are in correlation with Succi et al. [29] who demonstrated that the cultivation of bacteria with mannitol enhanced the survival of *Lactobacillus* strains. Similarly, Sathyabama et al. [44] showed that the incorporation of prebiotics into the alginate matrix during microencapsulation positively influenced the survival of probiotic bacteria, especially during storage at low temperatures. Furthermore, it has been shown that the joint application of alginate microspheres and prebiotics like FOS and gelatin may stimulate probiotic viability [41, 42] compared to cells encapsulated in alginate alone. According to our results, adjusting of the probiotic formulation composition allows adaptation of bacteria and leads to improved survival during freeze-drying, storage and digestion, which can be positively affected by the addition of EPS and selected prebiotic substrates. With the optimized probiotic formulation, the increased efficiency and biotechnological production of high-quality EPS-producing starter cultures or probiotics should be achieved.

## Conclusion

This paper describes the identification and functional annotation of the *eps* cluster involved in the biosynthesis of EPSs of *L. fermentum* D12. The functional potential of the producer strain was evaluated in vitro. Overall, the characterization of adaptive features, such as the protective potential of D12 EPS that could promote both survival and persistence of probiotics in the host GIT, is essential to clarify its health-promoting effects. Our results suggest the potential role of EPS in *L. fermentum* D12 functionality and the possibility of EPS to act as a potential trigger molecule, a characteristic that suggests that the D12 strain is, because of its dairy origin, a promising functional starter culture that produces EPS.

## Experimental procedures

### Bacterial strains and culturing conditions

The *L. fermentum* D12 strain was isolated from traditionally produced smoked fresh cheese of the Zagorje region, Croatia [45]. *L. plantarum* D13 was used as a non-EPS-producing strain. *L. fermentum* D12 and *L. plantarum* D13, were maintained as frozen stocks at − 80 °C in MRS (de Man Rogosa Sharpe; Difco, Detroit, MI, USA) while *S. aureus* 3048 and *L. monocytogenes* ATCC^®^19111™ were maintained in nutrient broth (Biolife, Milano, Italy), respectively, supplemented with 15% (v/v) glycerol and stored in Culture Collection of the Laboratory of Antibiotic, Enzyme, Probiotic and Starter Culture Technologies, Faculty of Food Technology and Biotechnology, University of Zagreb (CIM-FFTB). *L. fermentum* D12 was grown in MRS broth medium overnight at 37 °C. *S. aureus* 3048 and *L. monocytogenes* ATCC^®^19111™ were grown in nutrient broth at 37 °C overnight. Prior each experimental trial, strains were subcultured twice in appropriate fresh medium under corresponding growth conditions.

### Whole genome sequencing (WGS) and identification of putative gene clusters for exopolysaccharide (EPS) biosynthesis

Genomic DNA of *L. fermentum* D12 strain was extracted using a Maxwell^®^ DNA Cell kit in a Maxwell^®^ 16 Research System instrument (Promega, USA). Sequencing was performed on the Illumina MiSeq 2500 (Illumina, San Diego, CA) at IGA Technology Services (Udine, Italy) using a paired-end approach, as described in Banić et al*.* [25]. Contigs were classified as belonging to *L. fermentum* D12 when obtaining the best BLASTn v2.2.27 hit [46] in the NCBI nt database, where the whole genome sequence was submitted. The genome of *L. fermentum* D12 was uploaded to the web annotation service Rapid Annotations using Subsystems Technology (RAST; http://rast.nmpdr.org/rast.cgi) for automated annotation of sequenced genes, including those involved in EPS biosynthesis, followed by manual scanning [47]. This Whole Genome Shotgun project has been deposited at DDBJ/ENA/GenBank under the Accession no. RHMA01000000 (BioProject PRJNA388578, Biosample SAMN10319712). The version described in this paper is the first one. A circular map of the *L. fermentum* D12 genome was created using the DNA Plotter [48]. Furthermore, a BLASTp search against the NCBI protein (nr) database was performed.

### Characterization of EPSs produced by *L. fermentum* D12

#### Extraction and purification of EPSs

The *L. fermentum* D12 strain was grown on MRS agar plates for EPS production, as described in Kojić et al. [49]. Isolation of EPSs from *L. fermentum* D12 was performed using two different approaches to distinguish EPS released (EPS-r) into the culture medium from the EPS bound (EPS-b) to the cell surface. D12 strain was cultured in 400 mL of MRS broth supplemented with different carbon sources like glucose, galactose, lactose, fructose or sucrose 2% (w/v) at 30 °C for 2 days. Cells were separated from the broth by centrifugation at 7500*g* for 15 min at 4 °C.

The EPSs present in the supernatant (EPSs-r) were isolated according to Tallon et al*.* [50] with slight modifications. After centrifugation, the supernatants were treated with trichloroacetic acid (TCA) (Fisher Scientific, Waltham, USA) at a final concentration of 20% (w/v) for 30 min at 4 °C with gentle agitation. The precipitated proteins were removed from the samples by centrifugation at 8000*g* for 45 min at 4 °C and the supernatant was dialysed against water. EPSs were then precipitated from the dialysed supernatant upon the addition of four volumes of cold 95% ethanol, followed by overnight incubation at − 20 °C. The pellets obtained after centrifugation at 6000*g* for 30 min at 4 °C were dissolved in 2 mL of water and then dialyzed (cut off 10–14 kDa) first against 0.1 M NaCl and then against water. Finally, the purified EPSs-r were recovered by freeze-drying.

The isolation of EPSs bound to the cell surface (EPS-b) was performed according to Toba et al*.* [51] with slight modifications. The cells in the pellets from the first centrifugation, were washed with 250 mL of sterile 0.9% NaCl solution, and then centrifuged at 4000*g* for 30 min at 4 °C. The obtained pellets were suspended in 50 mL of sterile 1 M NaCl and sonicated at 550 W for 1 min (20 °C) in an ultrasound bath Elmasonic S 60 H in order to remove the potential EPS-b from the cell surface. Insoluble cellular materials were removed by centrifugation at 6000*g* for 30 min at 4 °C. EPS-b were purified from the supernatant as described above for the EPS-r samples and recovered by lyophilisation.

### General procedures

The presence of proteins and/or nucleic acids was determined by recording UV/Vis spectra in the wavelength range of 190–350 nm. Size exclusion chromatography was performed on a low-pressure chromatography system using a Sephacryl S-400 h column (1.6 cm i.d. × 90 cm) and 0.05 M NaNO_3_ as eluent with an 8.2 mL/h flow rate. Elution was monitored using a refractive index detector (Knauer, RI detector K-2301, Lab-Service Analitica) which was interfaced with a computer via PicoLog software. Samples were dissolved in 0.05 M NaNO_3_ at a concentration of 5.1 mg/mL and fractions were collected every 12 min. Fractions were pooled according to the elution profile, concentrated and dialyzed against water.

Analytical GLC was performed using a Perkin-Elmer AutosystemXL gas chromatograph equipped with a flame ionization detector and using He as the carrier gas. An HP-1 capillary column (Agilent Technologies, 30 m) was used to separate alditol acetates (temperature program: 3 min at 150 °C, 150–270 °C at 3 °C/min, 2 min at 270 °C), and partially methylated alditol acetates (PMAA) (temperature program: 1 min at 125 °C, 125–240 °C at 4 °C/min, 2 min at 240 °C). GLC-MS analyses were carried out on an Agilent Technologies 7890A gas chromatograph coupled to an Agilent Technologies 5975C VL MSD, using the same temperature programs reported above. For NMR spectroscopy experiments, the polysaccharides (~ 5 mg) were exchanged twice with 99.9% D_2_O by lyophilisation, dissolved in 0.6 mL of 99.96% D_2_O and introduced into a 5 mm NMR tube for data acquisition. Spectra were recorded on a 500 MHz VARIAN UNITY INOVA NMR spectrometer operating at 50 °C. Chemical shifts are expressed in ppm using acetone as an internal reference (2.225 ppm for ^1^H). NMR spectra were processed using MestreNova software.

### Composition and linkage analyses

For composition analysis, 0.5 mg of EPS-r2 were hydrolysed with 2 M trifluoroacetic acid (TFA) for 1 h at 125 °C. Subsequently, derivatization to alditol acetates was carried out according to Albersheim et al*.* [52]. Per-methylation was performed on 0.5 mg of lyophilized samples following the protocol of Harris et al. [53]. The per-methylated samples were hydrolysed with 2 M TFA for 1 h at 125 °C, and subsequently derivatised to alditol acetates to obtain a mixture of partially methylated alditol acetates (PMAA) which were analysed by GLC and GLC–MS. Integration values of the areas of the PMAA mixture were corrected by the effective carbon response factors [54] prior to calculating the relative molar ratios.

### In vitro functionality of *L. fermentum* D12

#### Antibiotic susceptibility testing

The antibiotic susceptibility of *L. fermentum* D12 was determined according to the breakpoints proposed for *Lactobacillus* by the European Food Safety Authority [22]. MIC in mg/mL for *L. fermentum* D12 was determined quantitatively using the E-test strips with a predefined gradient of antibiotic concentrations (bioMerieux, Marcyl’Etoile, France). The tested antibiotics included ampicillin, chloramphenicol, clindamycin, erythromycin, gentamicin, kanamycin, streptomycin, tetracycline and vancomycin. Briefly, inocula were prepared from an overnight grown *L. fementum* D12 in sterile saline to obtain a turbidity equivalent of 1 McFarland standard. MRS plates were inoculated with a prepared bacterial solution and incubated for 24 h at 37 °C. The diameters of the inhibition zones were measured and an average of three readings was calculated. MIC values were compared with EFSA guidelines for resistance cut-off values according to a genus [22].

### Adhesion assays

In vitro adhesion of *L. fermentum* D12 to the Caco-2 cell line was conducted as previously described by Banić et al*.* [25] with modification. Briefly, monolayers of Caco-2 were seeded into 24-well tissue culture plates (Falcon^®^; Durham, USA) until confluent monolayers were obtained and afterwards rinsed three times with phosphate buffered saline (PBS; pH 7.4). The cells of the overnight grown culture of *L. fermentum* D12 strain were washed twice with PBS (pH 7.4), and the optical density was adjusted to OD_620_ = 1. Afterwards, 1 mL of the prepared bacterial suspension (approx. 10^9^ CFU/mL) was added to each well and incubated for 1 h at 37 °C in an atmosphere of 5% CO_2_. The monolayers were then washed three times with PBS (pH 7.4) to remove unattached bacterial cells. The total number of adhered bacteria in each well was counted after lysing the Caco-2 cells with 0.05% (v/v) Triton X-100 (AppliChem, Darmstadt, Germany) solution at 37 °C for 10 min. To determine the number of viable bacteria, adhered to Caco-2 cells, appropriate dilutions in physiological solution were inoculated on MRS agar and colony counts were performed. The adhesion percentage was calculated using the following equation: (Adhered strains/strains added to the well) × 100. Experiments were repeated three times, and the results are reported as mean values of three individual experiments ± standard deviation.

*Lb. fermentum* D12 and *Lb. plantarum* D13 adherence to individual immobilized proteins of the ECM: fibronectin (BD BioCoat, Erembodegem, Belgium), type I collagen (Biocoat, UK), laminin (BD BioCoat, Erembodegem, Belgium) and mucin (Sigma-Aldrich, St Louis, MO, USA) was tested as described by Antikainen et al*.* [55] with modifications.

### Antibacterial activity testing

The soft agar overlay assay was used to test the antimicrobial activity of *L. fermentum* D12 against *S. aureus* 3048 and *L. monocytogenes* ATCC^®^19111™ [56]. Briefly, 5 μL of overnight *L. fermentum* D12 culture was spotted onto MRS agar (Biolife, Italy), and incubated at 37 °C for 24 h. Test-microorganisms were inoculated in 0.7% w/v nutrient soft agar (Biolife, Italy), and immediately poured onto D12 culture grown on MRS agar. The plates were incubated under optimal growth conditions. The ratio of the inhibition diameter (ID) to the spot culture diameter (CD) was calculated to determine the effective inhibition ratio (EIR) of *L. fermentum* D12 according to the following equation:

$${\text{EIR}} = \frac{{\left( {ID - CD} \right)}}{CD}$$

### Determination of GIT transit tolerance

Evaluation of in vitro survival of *L. fermentum* D12 during GIT transit was performed as described by Banić et al*.* [25]. Briefly, overnight cultures of the EPS-producing D12 strain were harvested by centrifugation (9000*g*; 10 min), washed twice, suspended in simulated gastric juice (pH = 2) and incubated for 2 h. Immediately after incubation in simulated gastric juice, the cells were harvested by centrifugation, suspended in simulated small intestinal juice (pH = 8) and incubated for 4 h. Viable cell counts were determined before, and after incubation in simulated gastric juice and small intestine juice using a standard pour-plate method on MRS agar and the values were expressed as colony forming units (CFU/mL). The experiment was repeated three times, and the results were reported as mean values of three individual experiments ± standard deviation.

### Alginate microencapsulation and freeze-drying

Microencapsulation of D12 cells in sodium alginate was performed according to Gbassi et al. [57] with slight modifications. The overnight grown D12 culture was pelleted by centrifugation at 4000*g* for 10 min, washed twice and suspended in 2% (w/v) sodium alginate (Fluka, Switzerland) with three different prebiotic substrates; inulin with fructooligosaccharides (FOS) (Magdis, Sveta Nedjelja, Croatia), mannitol (Difco, USA) and lactulose (Calbiochem, USA) at the final concentration of 5% (w/v). Microspheres without prebiotics served as control. The bacterial suspension was dropped aseptically into 150 mL of 1% (v/w) solution of calcium chloride which caused the droplets to harden into microspheres. After 1 h, the alginate microspheres were separated by decantation and rinsed with 0.9% NaCl solution.

Entrapped *L. fermentum* D12 cells in alginate microspheres were released by homogenizing in 2% w/v sodium-citrate (Biovit, Varaždin, Croatia) for bacterial count determination. Furthermore, alginate microcapsules were suspended in 10% (w/v) skim milk (Fluka, Buchs, Germany), frozen at − 80 °C and freeze-dried in Martin Christ Alpha 1–2 LDplus freeze dryer (Osterode, Germany). The freeze-dried encapsulated D12 cells were stored at 4 °C in closed glass containers. At predefined time intervals, the freeze-dried encapsulated D12 cells were analysed for their viability by the pour-plate method. Initial viability after freeze-drying was compared to the viability after 2 weeks and 1, 2, 3, 6 and 12 months of storage. Freeze-dried free cells and sodium alginate encapsulated D12 cells served as controls.

Free *L. fermentum* D12 cells and non-EPS-producing *L. plantarum* D13 cells were also freeze-dried after the addition of crude EPS extracts at different concentrations (0.2, 0.5 and 1.0 mg/mL) to evaluate their lyoprotective ability. Also, four different lyoprotectants lactose, inulin, sorbitol and sucrose at a typically used concentration of 10% (v/w) were applied individually to D12 and D13 cells. Obtained data of viable cell count after freeze-drying are expressed as cell mortality-a logarithmic reduction of the colony-forming units per gram of lyophilized cells powder (Δlog CFU/g). Subsequently, free and microencapsulated lyophilized D12 cells were exposed to simulated GIT conditions as described in “Determination of GIT transit tolerance” section.

### Scanning electron microscopy (SEM) analysis

To observe the EPS on the cell surface produced in situ, bacterial surface structures of *L. fermentum* D12 were visualized by SEM VEGA3 TESCAN (DET: SE; SEM HV: 10 kV). The SEM sample imaging was performed at the Center of Excellence for Advanced Materials and Sensing Devices, Institute of Physics, Zagreb. Sodium alginate entrapped *L. fermentum* D12 cells and purified EPS were subjected to SEM analysis. The samples were mounted on a metal stub. Images were acquired at a resolution of 200 μm for the EPS sample and at a magnification of 88× and 130× respectively. The D12-containing microspheres and the morphology of the EPS were observed at SEM, with an accelerated voltage of 10 kV.

### Statistical analysis

Results are expressed as means of three independent trials ± standard deviation (SD). Statistical significance was appraised by one-way analysis of variance (ANOVA). Pairwise differences between the means of groups were determined by the Tukey HSD test for post-analysis of variance pairwise comparisons (http://vassarstats.net/).

## Supplementary Information

**Additional file 1: Figure S1.** Circular phylogenetic tree based on whole-genome sequences showing the relatedness of *L. fermentum* strains.

**Additional file 2: Figure S2.** Comparison of the *eps* gene cluster found in *L. fermentum* D12 with those of other *L. fermentum* strains with the similar structural organization. The arrows indicate the genes with their orientation and in colors the functions in the *eps* cluster. The gray scale indicates the percentage of identity after performing tBLASTx, twisted gray areas indicate the alignment between opposite strands. The figure was done with Easyfig [58].

**Additional file 3: Figure S3.** a) Macroscopic observation of the ropy phenotype of *L. fermentum* D12 colonies grown on MRS agar. Scanning electron micrograph of the porous structure of the crude freeze-dried exopolysaccharide (EPS) extract produced by *L. fermentum* D12 after cultivation in MRS broth additionally supplemented with 2% w/v glucose (130×).

**Additional file 4: Figure S4.** Adhesion of *L. plantarum* D13 to extracellular matrix proteins in the presence of increasing concentrations of D12-EPS (0, 0.2, 0.5; and 1 mg/mL). *Bars significantly different (P < 0.01) from the untreated control.

**Additional file 5: Figure S5.** Scanning electron microscopy (SEM) of the microsphere of alginate entrapped *L. fermentum* D12 cells; SEM magnification 88×.

**Additional file 6: Table S1.** Genes of *L. fermentum* D12 involved in exopolysaccharides (EPSs) production and in the activation of the precursor molecules.

## Data Availability

The datasets used and/or analysed during the current study are available from the corresponding author on reasonable request.
