# Supplementary figures and images for: Lyophilized alginate-based microspheres containing Lactobacillus fermentum D12, an exopolysaccharides producer, contribute to the strain’s functionality in vitro
Source: Microb Cell Fact. 2021 Apr 17;20:85. doi: 10.1186/s12934-021-01575-6 (PMC8052780; doi:10.1186/s12934-021-01575-6)

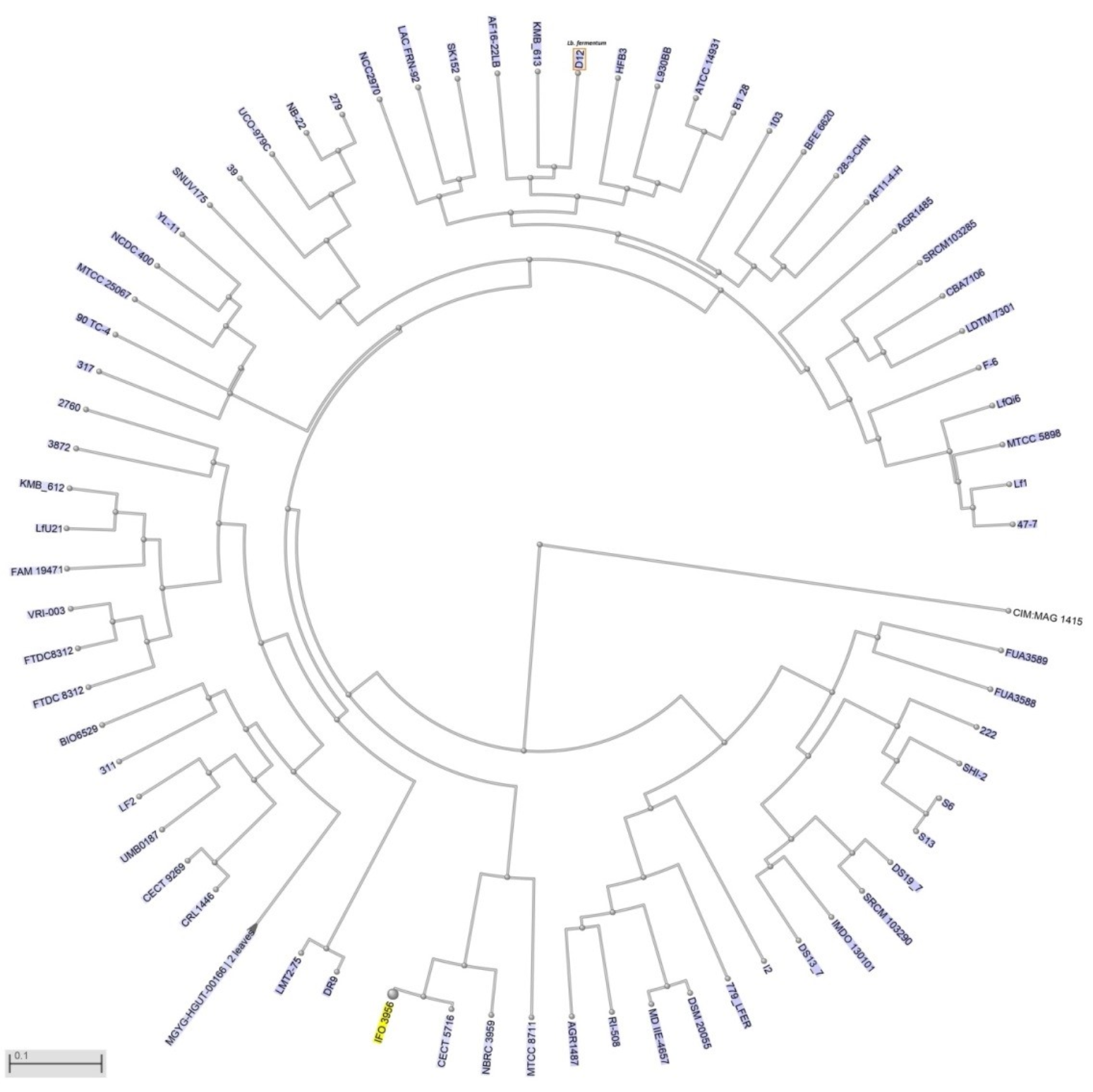

Supplement: Supplementary file 1 — Additional file 1: Figure S1. Circular phylogenetic tree based on whole-genome sequences showing the relatedness of L. fermentum strains. [file 12934_2021_1575_MOESM1_ESM.png]

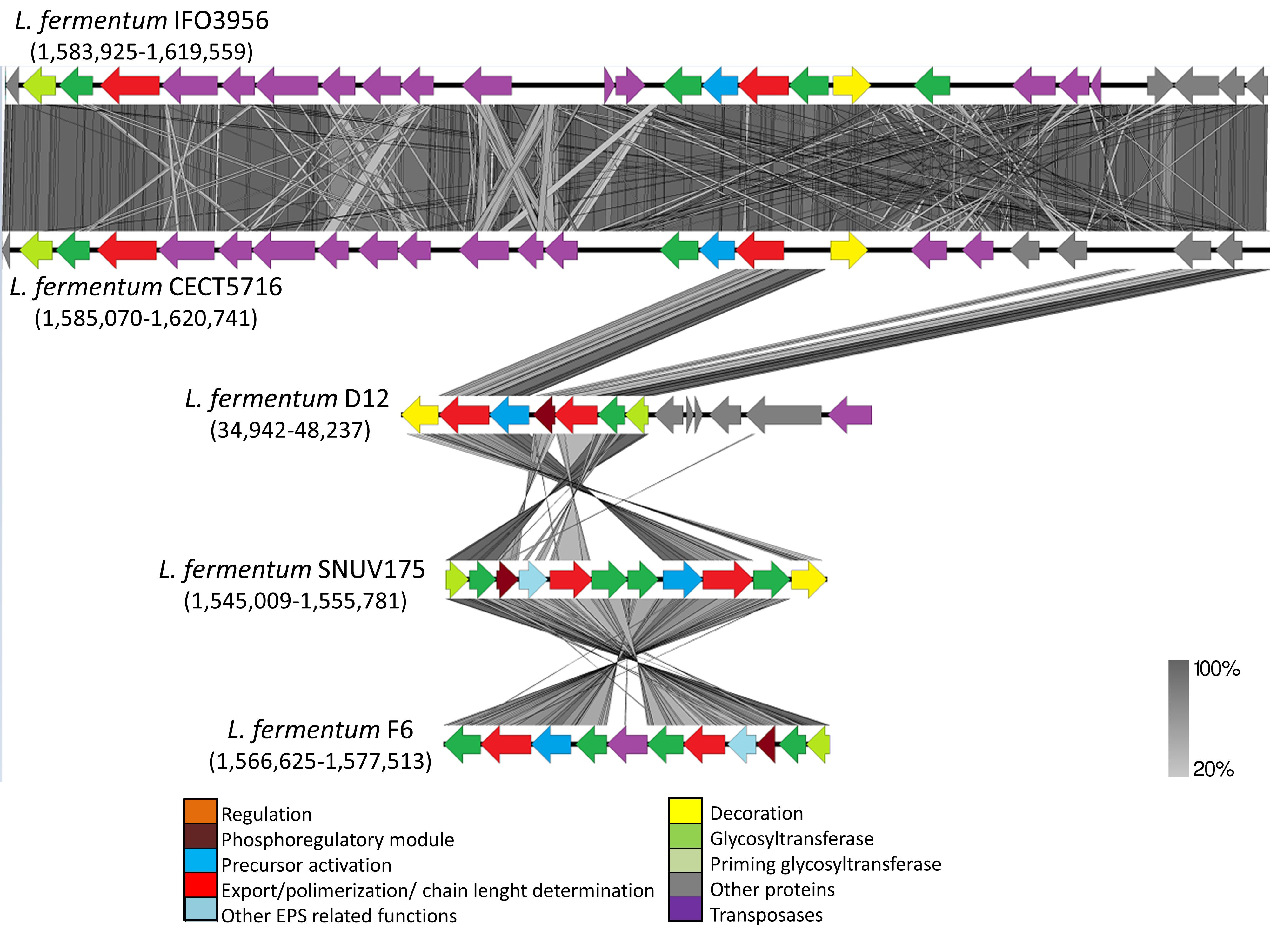

Supplement: Supplementary file 2 — Additional file 2: Figure S2. Comparison of the eps gene cluster found in L. fermentum D12 with those of other L. fermentum strains with the similar structural organization. The arrows indicate the genes with their orientation and in colors the functions in the eps cluster. The gray scale indicates the percentage of identity after performing tBLASTx, twisted gray areas indicate the alignment between opposite strands. The figure was done with Easyfig [58]. [file 12934_2021_1575_MOESM2_ESM.tif]

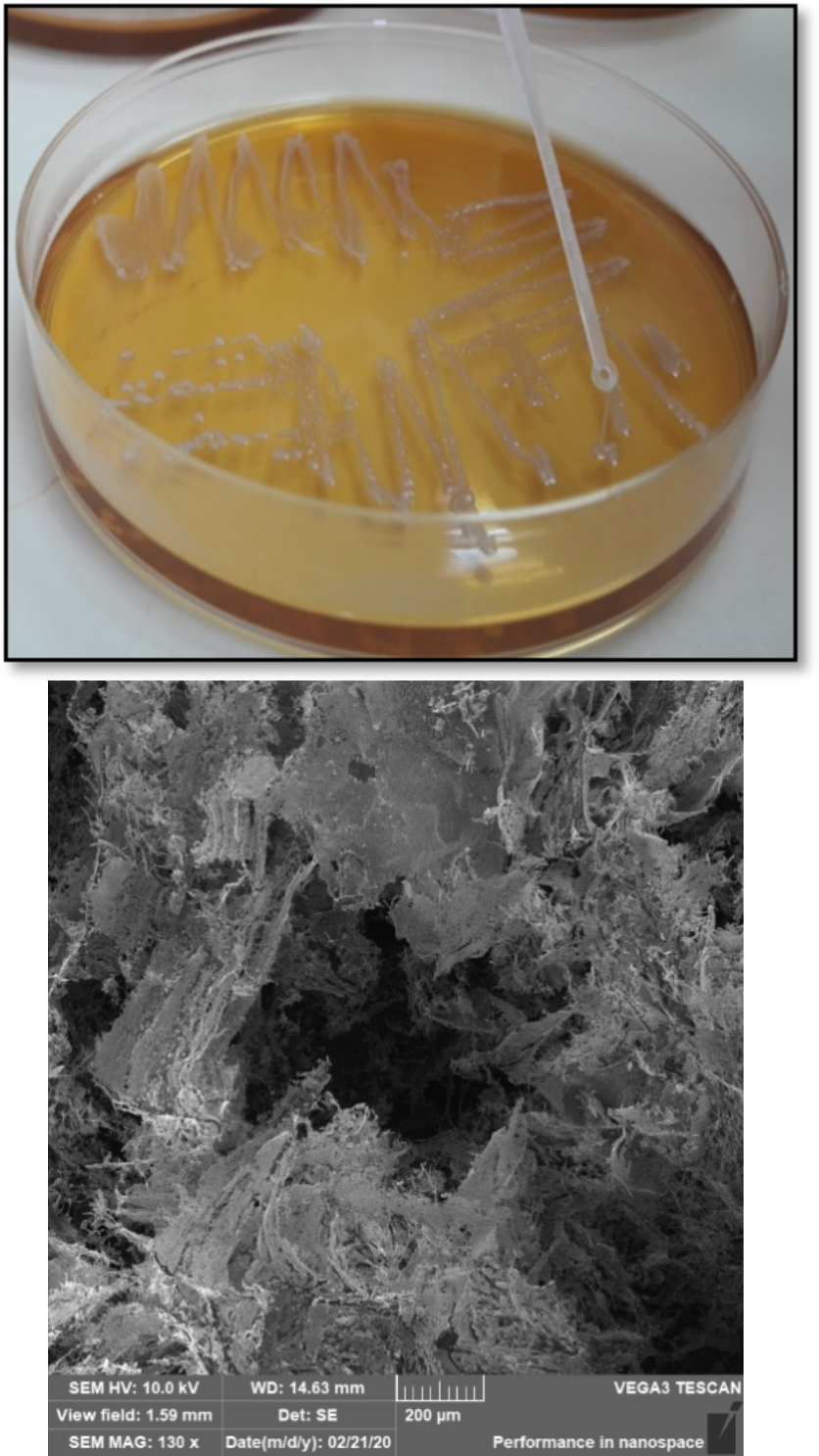

Supplement: Supplementary file 3 — Additional file 3: Figure S3. a) Macroscopic observation of the ropy phenotype of L. fermentum D12 colonies grown on MRS agar. Scanning electron micrograph of the porous structure of the crude freeze-dried exopolysaccharide (EPS) extract produced by L. fermentum D12 after cultivation in MRS broth additionally supplemented with 2% w/v glucose (130×). [file 12934_2021_1575_MOESM3_ESM.png]

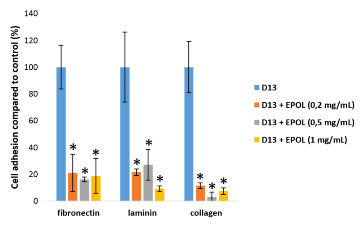

Supplement: Supplementary file 4 — Additional file 4: Figure S4. Adhesion of L. plantarum D13 to extracellular matrix proteins in the presence of increasing concentrations of D12-EPS (0, 0.2, 0.5; and 1 mg/mL). *Bars significantly different (P < 0.01) from the untreated control. [file 12934_2021_1575_MOESM4_ESM.tif]

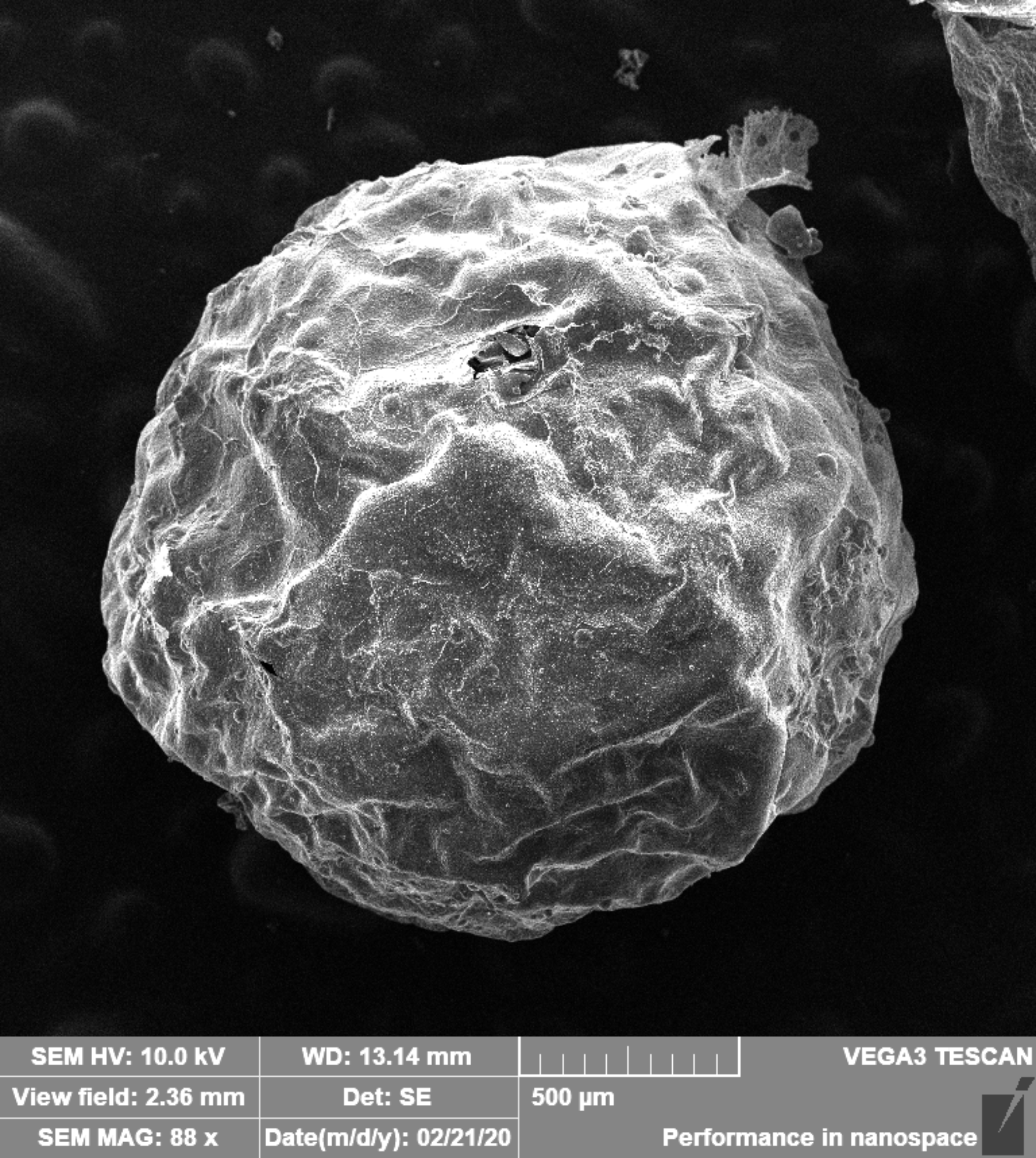

Supplement: Supplementary file 5 — Additional file 5: Figure S5. Scanning electron microscopy (SEM) of the microsphere of alginate entrapped L. fermentum D12 cells; SEM magnification 88×. [file 12934_2021_1575_MOESM5_ESM.png]
