# Supplementary material for: Lyophilized alginate-based microspheres containing Lactobacillus fermentum D12, an exopolysaccharides producer, contribute to the strain’s functionality in vitro
Source: Microb Cell Fact. 2021 Apr 17;20:85. doi: 10.1186/s12934-021-01575-6 (PMC8052780; doi:10.1186/s12934-021-01575-6)
Supplement: Supplementary file 6 — Additional file 6: Table S1. Genes of L. fermentum D12 involved in exopolysaccharides (EPSs) production and in the activation of the precursor molecules. [file 12934_2021_1575_MOESM6_ESM.docx]

**Supplementary table 1.** Genes of *L. fermentum* D12 involved in exopolysaccharides (EPSs) production and in the activation of the precursor molecules.

| **Gene name** | **LOCUS_TAG** | **Best BLAST match**  **product and source microorganism** | **Length**  **(aa)** | **%**  **identity** | ***e*-value** | **% Coverage** |
| --- | --- | --- | --- | --- | --- | --- |
| Exopolysaccharides synthesis | | | | | | |
| *epsA* | GW747_RS03535 | LCP family protein [*Limosilactobacillus fermentum*],  WP_003685760.1 | 333 | 99.10 | 0.0 | 100 |
| *epsC* | GW747_RS03540 | exopolysaccharide biosynthesis protein [*Limosilactobacillus fermentum*], WP_124890989.1 | 256 | 97.66 | 0.0 | 100 |
| *epsD* | GW747_RS03545 | polysaccharide biosynthesis tyrosine autokinase [*Limosilactobacillus fermentum*], WP_187703811.1 | 246 | 96.75 | 2e-173 | 100 |
| *epsB* | GW747_RS03550 | MULTISPECIES: exopolysaccharide biosynthesis protein [Lactobacillaceae], WP_035437027.1 | 256 | 100 | 8e-152 | 100 |
| *-* | GW747_RS07655 | acyltransferase [*Limosilactobacillus fermentum*], WP_023466034.1 | 352 | 99.72 | 0.0 | 100 |
| *wzx* | GW747_RS07660 | flippase [*Limosilactobacillus fermentum*], WP_004563040.1 | 472 | 99.79 | 0.0 | 100 |
| *epsC´* | GW747_RS07670 | LPS biosynthesis protein [*Limosilactobacillus fermentum*], WP_023466033.1 | 205 | 99.51 | 4e-145 | 100 |
| *wzy* | GW747_RS07675 | polymerase [*Limosilactobacillus fermentum*], WP_111523425.1 | 407 | 99.75 | 0.0 | 100 |
| *gt* | GW747_RS07680 | MULTISPECIES: DUF4422 domain-containing protein [Lactobacillaceae], WP_048340342.1 | 253 | 99.60 | 0.0 | 100 |
| *epsE* | GW747_RS07685 | sugar transferase [*Limosilactobacillus fermentum*], WP_004563036.1 | 224 | 100 | 9e-158 | 100 |
| Activation of precursor molecules | | | | | | |
| *galE* | GW747_RS00365 | UDP-glucose 4-epimerase GalE [*Limosilactobacillus fermentum*] (EC 5.1.3.2), RGW57828.1 | 331 | 99.70 | 0.0 | 100 |
| *galT* | GW747_RS09600 | Galactose-1-phosphate uridylyltransferase [*Lactobacillus fermentum*] (EC 2.7.7.10), AOR74297.1 | 485 | 99.59 | 0.0 | 100 |
| *galU* | GW747_RS00590 | UTP-glucose-1-phosphate uridylyltransferase GalU [*Limosilactobacillus fermentum*] (EC 2.7.7.9) WP_021349633.1 | 303 | 99.67 | 0.0 | 100 |
| *pngm/pmm* | GW747_RS00660 | phosphoglucomutase/phosphomannomutase, alpha/beta/alpha domain II [*Limosilactobacillus fermentum* ATCC 14931] (EC 5.4.2.8), EEI22570.1 | 577 | 99.65 | 0.0 | 100 |
| *glf* | GW747_RS07665 | UDP-galactopyranose mutase [*Limosilactobacillus fermentum*] WP_128492410.1 | 373 | 99.73 | 0.0 | 100 |
| *rfbB* | GW747_RS07590 | dTDP-glucose 4,6-dehydratase [*Limosilactobacillus fermentum*] (EC 4.2.1.46), WP_130125615.1 | 310 | 99.68 | 0.0 | 100 |
